# Co-producing a peer-led health conversation tool for the health services workforce to facilitate safer use of anabolic–androgenic steroids

**DOI:** 10.1186/s12954-025-01356-z

**Published:** 2025-11-24

**Authors:** Timothy Piatkowski, Sonya Weith, Emma Kill, Brooke Walters, Geoff Davey, Cameron Francis, Steph Reeve, Ross Coomber, Jason Ferris, Monica Barratt, Cheneal Puljevic, Emily Stockings

**Affiliations:** 1 School of Applied Psychology and Centre for Mental Health, Griffith University, Brisbane, QLD Australia; 2 Centre for Health Services Research, University of Queensland, Brisbane, QLD Australia; 3 Queensland Injectors Voice for Advocacy and Action, Brisbane, QLD Australia; 4 The Loop Australia, Brisbane, Australia; 5 Queensland Injectors Health Network, Brisbane, Australia; 6 Department of Sociology, Social Policy and Criminology, Faculty of Humanities and Social Sciences, University of Liverpool, Liverpool, UK; 7 Social Equity Research Centre and Digital Ethnography Research Centre, RMIT University, Melbourne, VIC Australia; 8 National Drug and Alcohol Research Centre, UNSW Sydney, Sydney, NSW Australia; 9 School of Public Health, The University of Queensland, Brisbane, QLD Australia; 10 The Matilda Centre for Research in Mental Health and Substance Use, The University of Sydney, Sydney, Australia

**Keywords:** Anabolic–androgenic steroids, Harm reduction, Health services, Image and performance enhancing drugs, Lived-living experience, Peers

## Abstract

**Background:**

Illicit anabolic–androgenic steroid (AAS) use poses physical and psychosocial risks. These issues are exacerbated by inadequate public health responses and well-meaning yet inadequately trained health workers. This study presents the development of a collaboratively designed *health conversation tool,* which equips health workers’ with a number of questions and strategic information to promote well-informed use for people who use AAS.

**Methods:**

This qualitative, multi-stage study co-produced a health conversation, a guided conversation tool that aims to incorporate a brief assessment, knowledge provision, sharing of harm reduction strategies and advice for health workers engaging with people who use AAS in community settings. The iterative co-design process followed a five-stage pedagogical approach (ideation, planning, creation, programming and sharing), guided by semi-structured interviews with 25 people who use AAS and a workshop focus group of 6 experienced AAS peers for further refinement. Guided discussion included exploration or harms, health enhancement and required resources. Qualitative responses were synthesised via inductive analysis to identify key themes, from which the conversation was developed and then tested with the AAS peer group.

**Results:**

There were two core findings from this research. Firstly, insights gained from people who use AAS in the key current practices related to safer use of these drugs. Secondly, based on these insights, a health conversation tool was co-produced, which encompasses a brief assessment, gauging people’s experiences with AAS, confidence in their knowledge, and support systems. The tool provides health workers with a suite of harm reduction strategies to offer to people who use AAS, such as proper injection techniques, considerations regarding their usage strategy, and suggestions for health monitoring.

**Conclusions:**

The collaborative design process ensured the health conversation tool reflected the lived-living experiences and priorities of people who use AAS, specifically fostering trust and engagement. This peer-driven approach filled gaps in harm reduction services, promoting informed decisions regarding AAS use as well as some health strategies. Expanding the peer workforce and integrating digital platforms can enhance the reach and sustainability of tailored harm reduction interventions for AAS and other communities.

**Supplementary Information:**

The online version contains supplementary material available at 10.1186/s12954-025-01356-z.

## Introduction

Anabolic–androgenic steroids (AAS) are a class of drugs that are structurally similar to the male sex hormone, testosterone, and produce effects by binding to the androgen receptor. While there are some medical applications, people use AAS non-medically for motivations like enhancing muscle mass, improving physical recovery, and boosting subjective wellbeing [1–3]. Illicit AAS consumption is known to come with several psychosocial risks such as acne vulgaris, hypertension, cardiomyopathy and an increased risk of mental disorders and other substance use [4]. This is important as we know, globally, 6.4% of men [5] and 4% of women [6] are exposed to these significant health risks. For people who use AAS non-medically, these risks are significantly compounded by the uncertainty about the content and quality of the substances available [7–11]. The challenges are further exacerbated by inadequate public health responses [12, 13] and poorly developed frameworks for health and harm reduction among this group [14]. One of the most pressing issues is the inadequacy of the current health workforce in addressing AAS use and harms, including general practitioners, pharmacists, and harm reductions workers, which is largely untrained and uneducated in addressing the specific needs of people who use AAS [15–17]. Research has found that health workers who work at needle syringe programs report challenges with engaging in meaningful harm reduction conversations with people using AAS compared to other groups of substance consumers [17], leaving this community underserved and at greater risk [15, 18–20]. In the case of AAS, a specialised health engagement strategy is critical to bridging gaps in workforce knowledge and ensuring that harm reduction practices are accessible and effective. This study presents the development and end-product of a ‘health conversation tool’, that aims to address both the needs of the workforce and the AAS community. By providing structured guidance, the health conversation tool equips health workers at needle syringe programs and harm reduction services to engage with people who use AAS.

## Background

AAS can be consumed through various methods, including oral ingestion, transdermal patches, and gels [21]. However, injecting remains the most prevalent method, particularly among people using AAS non-medically [22]. This method is favoured for its efficiency and long-lasting effects but carries risks, most notably, the transmission of blood-borne viruses like hepatitis C, and associated injection-related harms [23–25]. Research indicates that people who inject AAS often refrain from seeking medical attention when experiencing harm, largely due to their perception that medical professionals lack sufficient expertise in AAS use and related health issues, such as diminished libido and abnormal blood test results [26]. Stigma surrounding drug use, which includes AAS use, encompasses labelling, stereotyping, and discrimination, is often reinforced by public perceptions and media narratives that portray people who use drugs negatively [27]. This stigma is particularly problematic in healthcare settings, where people who use AAS often face judgment and a lack of understanding from medical professionals [28]. As a result, people who use AAS may delay seeking treatment for AAS-related effects, which can exacerbate health risks [29]. Although there is some receptivity among people who use AAS to engage with health workers (e.g., at needle and syringe programs) these workers often report feeling unprepared to address the specific needs of this group [15, 17, 30]. As such, there is an emerging demand for enhanced training on AAS use for healthcare workers to better serve this distinct and growing client population. In some nations like Australia and the United Kingdom, people who use AAS make up a substantial proportion of needle and syringe program clientele [31, 32] with new initiates of AAS injecting (injecting for less than three years) increasing from 18% in 2019 to 26% in 2023 [33]. In the United Kingdom, although 36% of people who use AAS report adverse symptoms in the past year only 17% seek help [34]. Given these new initiates to injecting have a level of relative inexperience of using AAS and managing their health, thus, there is a growing need for increased harm reduction efforts to safeguard the health of this expanding population.

To mitigate these challenges, there have been calls for initiatives focused on reducing stigma and enhancing consumer knowledge to better support people who use AAS [27, 35, 36]. We note that the ongoing stigma against people using AAS not only marginalises them but also perpetuates inequitable access to healthcare, denying them the same level of care afforded to others, contributing to undue harms. Kate Seear [37, 38] has previously suggested that this type of discrimination violates the basic human rights of people who use drugs, as it hinders their ability to receive appropriate support and exacerbates health disparities within this population. Research has shown that interactions with healthcare professionals are often inflected with stigma and pathologisation, which can discourage people who use AAS from seeking care or engaging with health advice [39, 40]. Nourse et al. have recently highlighted that notions of health, risk, and harm are contested within this community, and that peer-led or trusted interactions can be more influential than traditional medical advice [41]. Therefore, particularly for people who use AAS, there is an urgent need to prioritise health responses to harm reduction which are salient and engage this community. Despite the growing need, harm reduction information specifically tailored to AAS use remains scarce for both people who use AAS and the health workforce [17]. As Bates and Vinther have suggested [42], there is a clear need for targeted interventions that support this cohort, and we believe the health workforce in uniquely positioned to assist with this.

Drug checking services provide critical analysis and health information for illicit substances, helping to reduce risks and promote safer use among people who use drugs. AAS testing presents unique challenges due to the complex chemical composition these drugs, making detection and analysis difficult [9]. There have been some jurisdictions (e.g., Australia, Switzerland) which have operated services catering to people who use AAS specifically [7, 43, 44]. These pioneering services provides critical analysis and health information to people who use AAS, aiming to reduce risks and promote safer practices. However, a key limitation has been the absence of an evidence-based brief health intervention which can be delivered with individual test results. It is also important to ensure the development of targeted educational interventions and prevention messages resonate with this diverse cohort [26, 45, 46]. Studies suggest that this group often turns to social networks involving peers with lived-living experience for advice, who are often perceived to be more preferred and trusted options for advice regarding usage [47, 48]. Considerations of alliances between quality medical care and information through public health systems remain tarred by repeated instances of inadequate knowledge [17, 26, 49] and limited receptivity among people who use AAS to education offered by professionals lacking lived experience [26].

Brief health interventions involve screening and assessing all patients for alcohol or drug use, enabling clinicians to provide information and advice to mitigate associated harms [50]. These interventions are opportunistic, targeting individuals identified through routine screenings rather than those actively seeking help. They are most effective for individuals at risk of developing harms, rather than those already dependent or experiencing severe drug-related issues. Recent concerns have been raised about their real-world effectiveness, highlighting the need for repeated sessions rather than relying on a single encounter. Our study introduces a ‘*health conversation tool*’ that equips health workers with peer-led strategies to reduce AAS-related harms, demonstrating potential to enhance help-seeking behaviours. We draw on the concept of a brief health intervention with the creation of the ‘tool’, which represents a type of brief intervention, whereby health workers offer evidence-based suggestions regarding strategies to reduce the harms which drugs can cause. The lead author initiated a program of work aimed at developing a health conversation that could accompany the provision of AAS test results. This health conversation tool was designed to equip health workers with the necessary tools to engage people who use AAS in meaningful discussions about AAS harm reduction. Given the lack of integration for health workers with lived-living experience of AAS use presently, the strong reliance on people who use AAS to seek information from their peers [15, 18, 51–54], and the unique insights provided by peers with lived-living experience, ‘steroid peers’ are vital in offering tailored advice and guidance to service-users [55]. The aim of this study was to adopt a co-production process to develop a health conversation tool and present it as a resource that can be disseminated across multiple platforms. In doing so, we seek to provide equitable harm reduction services for people who use AAS, addressing a critical gap in current harm reduction efforts and ensuring that this community receives the same level of support as those using other substances.

### Approach

The concept of co-production was first introduced by economist Elinor Ostrom in the late 1970s to describe a process where contributions from people outside a given organisation are used to create goods and services, including the assessment, management, and delivery of public services by both users and providers [56]. This idea was later expanded by scholars and activists to encompass principles of social justice [57]. Currently, co-production is a salient concept in policymaking, governance, and a diverse array of domains, including health and harm-related behaviours and is broadly accepted as adding value and justice [58–61]. It represents a collaborative approach aimed at improving health outcomes through the development of ‘peer-led’ and people-centred services [55]. Without input and guidance from those with lived-living experience, we miss important situational and contextual cues and personal narratives that help to inform the needs of the people [62]. Therefore, the aim of this research is to present the development of a co-produced health conversation tailored for people who use AAS, aimed at supporting informed decision-making and promoting harm reduction.

## Methods

### Design

This study is part of a larger overarching study aimed at improving health outcomes for people who use AAS through enhanced harm reduction strategies. This research represents a qualitative collaborative study focusing on the co-production of a health conversation for health workers to utilise to engage with people who use AAS. Ethical approval was granted from the Griffith University Human Research Ethics Committee (Approval: 2023/784).

### Recruitment

Participants were recruited through established community networks, including Queensland Injectors Voice for Advocacy and Action (QuIVAA) steroid advisory group [63]. QuIVAA has longstanding relationships with people who use AAS and ensured recruitment was conducted in a culturally safe and trusted environment [44]. Participants were approached through service promotion and peer outreach channels, reflecting purposive sampling aimed at including a diverse range of experiences with AAS use.

### Procedure

**Fig. 1:**
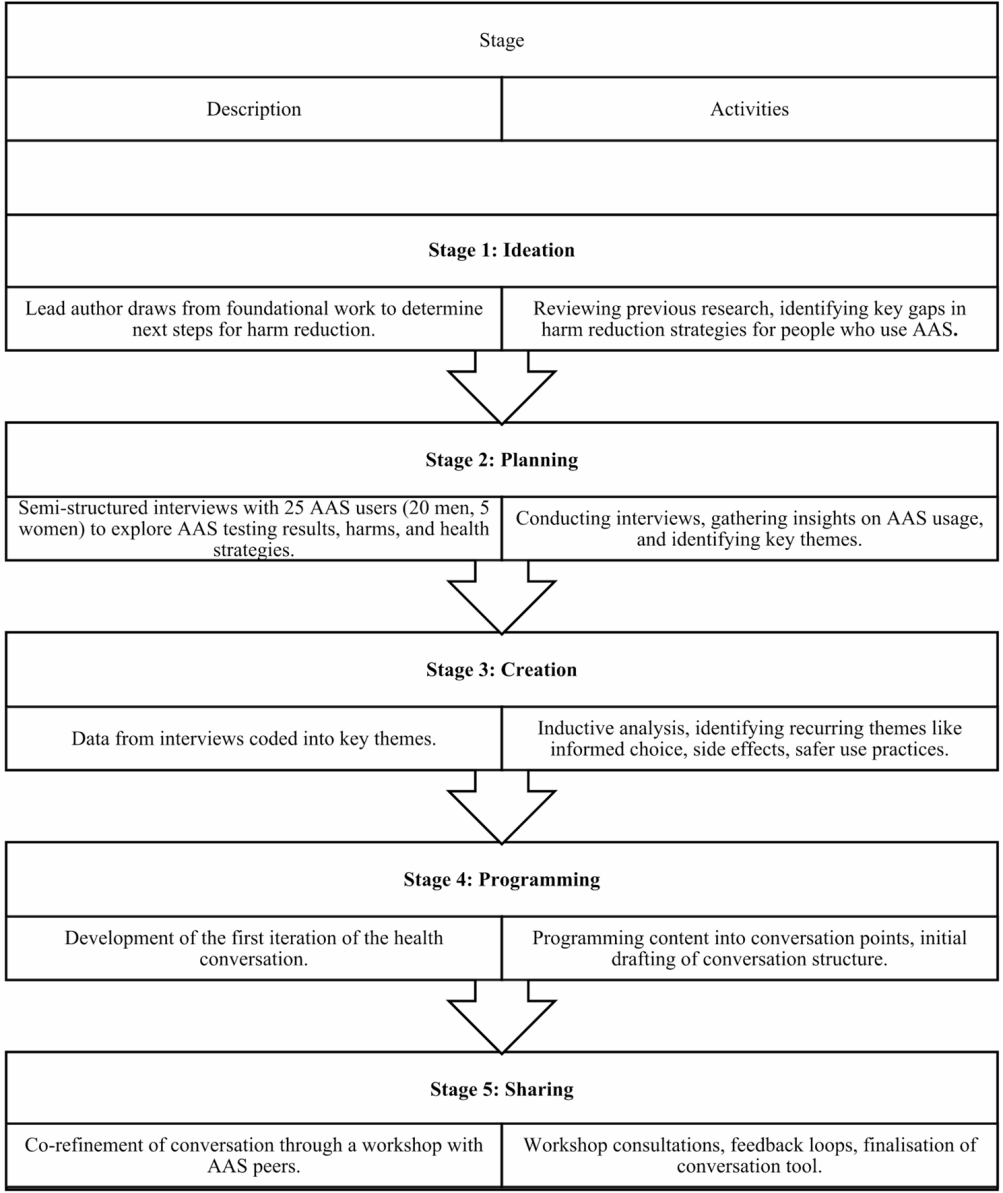
Collaborative development process for health conversation tool

A multi-stage collaborative process [64] assisted us to produce a tailored *health conversation tool* for people who use AAS, to be delivered by a health worker in a needle service provision setting during face to face interaction (see Fig. 1). To map this process from its inception the lead author used a five-stage pedagogical approach (ideation, planning, creation, programming, sharing) [65]. This ideation stage began by the lead author drawing on his foundational work [10, 11] to consider the next important steps for harm reduction among this community. This was subsequently followed by the planning and creation phases which began in June–August 2024, with 25 people who use AAS (20 men and 5 women; *Median age* = 32) participating in semi-structured interviews (*Median length* = 50 min). These interviews centred on AAS testing results from the overarching project which informed this work. The interviews examined broader AAS usage practices, including associated harms and health enhancement strategies. A significant portion of the discussion focused on identifying the support and resources necessary to facilitate health improvement and harm reduction. Participants were asked specific questions, such as: How confident are you in your ability to change your AAS usage based on the new information provided? What support or resources would help you in making any desired changes? How serious do you consider the potential health risks associated with using AAS? Do you believe you are at risk of experiencing negative health effects from using anabolic–androgenic steroids (AAS)—Why or why not? The participant insights were pivotal in shaping the planning and creation phases.

Data obtained during the creation phase was concurrently utilised in the programming phase through use of inductive analytic techniques for qualitative data, completed by lead author [66]. Codes and categories were identified by reviewing participant responses, focusing on frequently mentioned or significant topics, and extracting key categories as they emerged (*see *Table 1). Participants identified key areas of importance (i.e., physical and mental side effects, and safer use practices), which the lead author combined with his knowledge of extant literature pertaining to AAS harms and his own lived-living expertise. This peer-expert lens guided the subsequent phases of programming and sharing from categories into conversation points. The first iteration of the *health conversation tool* was designed to address the resultant key themes such as informed choice, non-judgmental dialogue, and specific health concerns of people who use AAS. In his role as a peer researcher, the lead author leveraged his experience in conducting bespoke, evidence-based training for government and non-government organisations, including workforces of medical practitioners, pharmacists, and health and harm reduction workers.

**Table 1:** Examples of inductive category development

Quote	Codes	Category
P20: Information about any compounds that I'm not familiar with. Whether it's side effects or benefits. Or how it can affect what systems it may affect 'cause I know are also affect kidneys, liver injectables generally it's just cardiovascular system, but there's also some injectables that affect your like neurological like tren and then all those kind of risks	Health risks	Physical and psychosocial risks
P8: The major barrier is not having a point, a trust, a trustworthy point of resource or information […] providing medical help needed in terms of blood testing and providing advice in both, direct and indirect, on reducing [use]. Allow them to do what they need to do, but providing the information that they need to look after themselves and giving them information that they given whatever outcome they're looking for, that they can get given better information	Health choices	Harm reduction
P12: […] my liver and my blood pressure and my cholesterol. So those are things that I keep very close monitoring on	Health checking	Health monitoring
P15: Some kind of resource 'cause like you have to go on forums and everything and if there was like one kind of site or or something that you could download and like you know click on it says this does this if you're going to do this I would be taking this because otherwise you just got to do a whole heap of research. If there's just one place you can go to that’s best	Health promotion	Safer use information
P14: I think it's [safe injecting] really one of the most important parts. Personally, for myself, for example, the stuff I told you about that I tried that was different, like my shoulder and arms was swelling up to double the size. Super red. Like they're gonna explode. And I was like fuck. Is this an infection?	Infection	Safe injecting
P1: I want to make sure that if I was getting a drug, it was going to be that drug	Compound quality	Testing
P13: I knew what they [AAS] were obviously, but I had no sort of idea four years ago, so. And most of my learning has been through open conversations with lived experience from mates who have used it right? So, if you don't have that you’re kinda flying blind	Lived experience	Peer-led

### Health conversation tool

Following the identification of key themes, expert members of the research team (EK, CF, and GD) reviewed and refined the health conversation materials [67] based on their extensive expertise working in the substance use health and harm reduction sectors (accumulated experience > 60 years). Based on these elements, the initial structure of the health conversation tool included a brief assessment of people’s experience and knowledge, strategies for harm reduction, and practical advice tailored to individual needs. Emphasis was placed on the health worker highlighting the importance of regular AAS testing, health monitoring, and providing resources for safer use. Additionally, it led to the establishment of a specialised "steroid area," an online resource housed in a drug harm reduction website named Hi-Ground. This is a dynamic website designed to promote health-enhancing and harm reduction practices for people who use image and performance enhancing drugs. This resource continues to adapt through ongoing consultation with people who use AAS, ensuring its relevance and accessibility.

The sharing phase comprised co-refinement of the conversation objectives [68] and planned logistics for a workshop session. The initial health conversation tool was then further developed via consultation with six experienced AAS peers (three women and three men; *median age* = 42)—including the lead author. The consultation, occurring in the format of an online, focus group focused on co-development, with the goal of collaboratively refining the tool. This workshop ran in September 2024 and was 75 min in length. The lead author recorded this workshop session and also took notes during this time. Following the workshop session, he applied and balanced the guidance and expertise of the peers with his knowledge of workforce needs. The final stage of the process involved a feedback loop, where peers reviewed the revised materials and were able to provide additional input to finalise the conversation structure.

The findings section presents the incorporation of the participants through workshop consultation. We share this process openly, alongside a version of the health conversation tool (see Supplementary Materials).

### Findings

The workshop feedback highlighted several important aspects for designing a health conversation specific to AAS. Firstly, education was viewed as a key tool, but the approach must be balanced—providing resources and accountability without trying to control behaviour. Secondly, participants described that there is a need for practical, trusted, and clear information on injection practices and risks, particularly around infections. They noted that resources are helpful but must be curated and centrally located within trusted services. Participants also highlighted that the health conversation must be concise, clear, and relevant to the person’s motivations, ensuring it fosters trust and positive engagement rather than resorting to fear-based approaches.

### Brief ‘check-in’

The conversation begins with an introductory statement which sets a collaborative tone and prepares clients for a detailed discussion about their AAS use. The initial section of the conversation involves a brief assessment to understand the person’s experience with AAS. Participants agreed that, particularly for the less-experienced people using AAS, there was a need to keep messaging clear and direct.

Simone [52, woman]: You really wanna make sure that those first-time users are not feeling as though they’ve been asked questions that they can’t answer and they don’t understand.

People are then asked about their history with these substances, ranging from first-time use to frequent use. This helps gauge their level of experience and adjust the conversation accordingly. Next, clients evaluate their confidence in their knowledge about AAS, with options ranging from "not confident" to "very confident." Lastly, people are asked about their support system, determining whether they feel they have adequate assistance for making health decisions. All people are offered the health conversation regardless of these responses.

### Harm reduction

Following the initial check-in and information gathering process, the conversation focuses on harm reduction strategies, which aligned with the community values of well-informed health enhancement with AAS.

Friedrich [32, man]: People will honestly do whatever they like…you can only give them the information and let them make the decision, they're adults at the end of the day.

Creating a supportive environment where people who use AAS feel safe to discuss their experiences and challenges is essential for addressing the psychosocial aspects of use.

Simone [52, woman]: And then maybe there's that space for them to feel safe enough to open up as well.

Doing so allows for tailored educational support based on their self-reported knowledge level. Peers acknowledged the limitations of education, suggesting that while it is essential to provide accurate information, ultimately, people will make their own choices regarding AAS use. This reflects a harm reduction approach, where the role of education is to produce well-informed decision making.

People who use AAS are invited to share their current harm reduction practices, such as whether they test their AAS. For those who do not test their substances, the conversation emphasises the importance of regular AAS checking and directs them to services and databases like PEDTest Australia [69, 70], ROIDCheck [71] and CanTEST in Canberra [72]. This ensures that people who use AAS are aware of and can access critical testing resources.

### Access to information

People who use AAS are asked about their current sources of information regarding AAS use. Those without adequate resources are directed to Hi-Ground, which offers comprehensive resources on AAS, including safe use practices and potential side effects.

Baruch [42, man]: You can’t just focus on gains; you have to know what’s happening in your body too. That’s where people slip up.

Peers endorsed the value of digital resources, emphasising that they empower people to take accountability for their health decisions.

Simone [52, woman]: The digital resource is good. It then gives people that chance to go and research for themselves and actually read and be accountable for the choices that they're also making.

By providing accessible information, people are encouraged to educate themselves further and make well-informed choices.

Trust in the service which people who use AAS are accessing was seen as pivotal to community engagement.

Simone [52, woman]: I think the fact that they're turning up […] shows that they trust in the service first and so having information available on the website of a service that they trust, they're more than likely to engage with that.

The peers believed that people attending the service would be more likely to engage with the health information provided. This represents a critical opportunity to build on this motivation and provide comprehensive harm reduction education.

### Health monitoring

One of the key practices that has emerged in the context of health monitoring for people who use AAS is blood checking [73]. Regular blood work can help people track their health markers, identify potential risks, and ensure safer usage practices [74]. Several peers also pointed out that some consultation services for blood work offer consultations for abnormal results, which could be valuable:

Baruch [32, man]: Does i-medical [online blood testing provider] offer [it] because I know Roidsafe [online blood testing provider] does. If you come back with abnormal results, they give you the option to consult with their doctor?

Building on this, another peer also emphasised the need to inquire about people’s health concerns first, before tailoring the information effectively:

Hannah [44, woman]: Might be worth asking, do they have health issues they’re worried about.

Peers recognised the need to balance the pursuit of physical results with a keen awareness of internal health. People may prioritise muscle gains over their health, which can lead to overlooking vital signs of harm. However, existing literature highlights that this behaviour is not solely due to lack of knowledge or neglect; Fraser et al. demonstrated that people who use AAS often want to engage with healthcare but face barriers due to stigma [39]. Additionally, Nourse et al. have shown that health encounters frequently constitute AAS consumers as ‘cheating’, deceptive, or unhealthy [40]. These structural and social factors shape health behaviours and cannot be reduced to individual choice alone.

Friedrich [42, man]: A lot of people don’t check their bloods or look at their health until something goes wrong. There should be more info about staying on top of that.

Many peers noted that many people who use AAS address their health once issues arise, which, when considered alongside previous scholars findings [41], should be interpreted in the context of stigma and constrained access to supportive healthcare rather than as an inherent lack of concern or knowledge. This highlights the need for health conversations to encourage regular monitoring, such as blood tests, as a preventative measure to avoid long-term health complications associated with AAS use. This section of the health conversation emphasises the importance of informed decision-making and offers people who use AAS practical resources to enhance their knowledge and safety.

The conversation also addresses the necessity of regular health monitoring. People are asked if they perform regular health checks, including blood work and blood pressure monitoring. An important piece of collaborative design at this point was in establishing temporality around the health checking process for people. One peer suggested a longitudinal approach to health checks to better understand users' health monitoring practices:

Simone [52, woman]: It might sound silly, but do you get, is in like your present tense? It could be good to ask, “have you ever [checked health markers]” so that way you can actually get the longevity as to what their health checks look like over their length of their kind of adult life.

Those who do not perform health checking are advised to seek regular monitoring through medical professionals or online services. Access to accurate and comprehensive information about services is crucial. Another peer suggested detailing available services:

Baruch [32, man]: Do you want to put down the services that are available for them, like in i-medical and Roidsafe?

### Safer use practices

The conversation then covers practical advice tailored to the method of AAS administration. For those using oral AAS, the conversation recommends keeping cycles short to minimise liver stress. Reflecting on that point one of the peers suggested informing people about the dangers of long-term cycles rather than just focusing on short-term usage:

Virginia [44, woman]: I would ask them if they are aware of the risks of danger over long term cycles rather than short, rather than just saying keep it short.

Based on this, and further discussions in the workshop, the health conversation also encourages people to consider their use within a framework of well-informed choices.

Hannah [44, woman]: Maybe instead of like, you know, telling him to reduce the use or whatever you just ask, are you having any negative side effects that are worrying you?

Incorporating this feedback meant people were consulted regarding their usage patterns, including whether they are taking breaks between cycles and having a usage strategy in place.

In the health conversation, people who inject their AAS are asked about the condition of their injection sites and are directed to local NSP services for safe injecting equipment and resources if needed. Concerns arose regarding infection, injection site safety, and the practical aspects of AAS injection. Peers discussed the necessity of discussing injection technique, risks, and safe practice.

Hannah [44, woman]: Do you have any injection sites that you're worried about or any possible infections you're worried about?

This underscored the importance of ensuring people who use AAS are engaged on issues of injection safety and infections, which are often overlooked but represent a major health risk for people who use AAS.

Hannah [44, woman]: That's the number one reason that a steroid user is going to end up in ED [emergency department] is from an infection.

By prompting this line of inquiry, the health conversation can open pathways to deeper engagement and potentially connect users to clinical support. Many AAS users may be unaware of the availability of free needle exchange services, and peers stressed the need to integrate harm reduction messaging into the steroid testing process. This includes information about needle exchange and safe injecting practices.

Hannah [44, woman]: Would it be worth sticking it on the list there of ‘Are you aware that you can get your needle exchanged here for free?’ Because a lot of steroid users may not be aware of that.

This comment underscored a knowledge gap among the community. Incorporating this information into the health conversation could significantly increase uptake of safe injecting supplies, thereby reducing the risks of infections and other complications. It also highlights the need for a proactive approach in delivering harm reduction information.

Simone [52, woman]: Just reminding people about things like rotating injection sites, using clean needles, and not sharing, it's basic stuff but people forget, or they don’t know.

Peers emphasised the importance of reinforcing harm reduction practices, such as rotating injection sites and using sterile equipment. Although these practices are simple, they are critical to preventing infections and complications [17]. The reminder to include these points in the health conversation reflects the peers’ recognition that even experienced people may neglect foundational safety measures.

Guidance is also provided on ensuring that time ‘off’ from AAS equals the time spent using them, and on having a post-cycle therapy (PCT) plan. PCT is a critical strategy used by people after completing a cycle of AAS to help restore the body’s natural hormone production [75]. This process typically involves the use of medications that stimulate the pituitary gland to resume testosterone production, minimising the negative effects of hormone suppression that can occur during AAS use [75–79]. By implementing a well-structured PCT plan, people report they can mitigate side effects and improve hormonal recovery [80], following their AAS cycle.

Baruch [32, man]: People know they should be doing PCT, but they don’t always know what that actually means or the risks of skipping it.

Peers identified a gap in community knowledge regarding PCT. While many people using AAS might be aware that PCT is essential, they often lack a deep understanding of its purpose or the risks associated with omitting it.

Virginia [44, woman]: There needs to be more info on how to do PCT properly, like what to take and when. A lot of guys just wing it.

This suggests the need for clear and accessible education on the importance of PCT in maintaining long-term health post-AAS use. Peers discussed that there are misconceptions among the community.

Virginia [44, woman]: There’s this misconception that once you stop using, everything goes back to normal, but that’s not always the case. Physically and mentally, there’s a long recovery period.

People often believed that discontinuing use will lead to an automatic return to baseline health, but peers pointed out that recovery, both physical and mental, can be prolonged and challenging. This highlights the importance of addressing post-cycle recovery in health conversations, ensuring users understand the need for ongoing care even after they stop using AAS.

For women, tapering use is recommended where possible. Generally, however, peers mentioned there was a lack of information for women.

Hannah [44, woman]: There’s not enough information out there for women. Most of what you hear is aimed at guys, and women just have to figure it out on their own.

Most of the peers noted the lack of tailored resources for women who use AAS, highlighting the male-centric focus in available harm reduction materials. This emphasises the need for gender-specific information in health conversations, acknowledging the unique experiences and risks for women using AAS.

Hannah [44, woman]: The risks for women are different, like virilisation. That needs to be made clearer because once those changes happen, there’s no going back.

Female-specific risks, such as virilisation (the development of male physical traits), were a significant concern for all peers, irrespective of gender. They expressed the need for more explicit information on the irreversible effects of AAS for women, which should be clearly communicated during health conversations to promote informed decision-making.

### Addressing physical and psychosocial aspects

The conversation concludes by addressing potential physical and mental health issues related to AAS use. Peers discussed how important it was to bring these concerns to surface and provide actionable moves forward.

Hannah [44, woman]: I know lots of people who are worried […] but too scared to get it checked.

Based on this feedback, people who use AAS are asked if they are experiencing any distress, such as physical symptoms or mental health concerns.

Virginia [44, woman]: I think it’s confronting, but I think it’s a really important aspect to be aware of as well.

Acknowledging that certain topics might be confronting but are important for people to be aware of is crucial for refining the approach to providing information and support.

Friedrich [42, man]: Steroids affect your body and mind. You can feel great physically but be struggling mentally, and no one really talks about that.

All peers highlighted the dual nature of AAS effects, impacting both physical and mental health. While people may experience physical benefits, such as increased muscle mass or strength, they may simultaneously face psychosocial challenges, including mood swings, aggression, or depression.

Hannah [44, woman]: A lot of guys talk about getting jacked, but no one wants to admit they’re dealing with stuff like anxiety or feeling down. That side of it gets ignored.

There is often a reluctance among people using AAS to openly discuss the psychological toll of AAS use [81]. This suggests that health conversations should create space for discussing mental health challenges, reducing stigma around the psychosocial aspects of AAS use and promoting mental wellbeing.

Those reporting issues are encouraged to seek support from peers, reassess their goals, and consult Hi-Ground resources for additional guidance. This section ensures that people receive appropriate support for any adverse effects they may be experiencing.

### Other feedback and refinement

Feedback from AAS peers played a crucial role in refining the health conversation tool, at an overarching level. However, this was also evident in specific alterations as well.

Adjusting language to be more precise and empathetic when discussing side effects was believed to improve the effectiveness of harm reduction strategies and service-user engagement. The health conversation tool was believed it would be most effective if it remained straightforward and focused on key issues to avoid overwhelming or confusing anyone from the community.

Baruch [32, man]: [Regarding health effects] I wouldn’t say “negative”, I’d say adverse because, yeah.

Peers advocated for a balanced approach, focusing on core issues like safe injecting, infection control, and the quality of substances used. While there is a broad range of information that could be covered in health conversations with people using AAS, there was consensus that keeping the conversation focused on key health risks would be most effective. Overloading people with information may reduce engagement or make the conversation less impactful.

Virginia [44, woman]: I think you overcomplicate it if you go further.

Peers valued the structured approach and the focus on non-judgmental dialogue and informed choice. The iterative design process also included further reviews after the workshop, which helped to tailor the conversation. The digital reviews were an essential component in refining the health conversation tool, allowing peers to provide further feedback and considerations on the next iteration. These reviews were useful in assisting the resource to be relevant and responsive to the lived-living experiences of people who use AAS. Peers highlighted they were satisfied with the tool. Others stressed the need for people using AAS for the first time to start with milder compounds, such as testosterone for men and the significance of monitoring cycle length and dosage to avoid adverse effects. This feedback helped further elevate the conversation by addressing issues like cycle timing, the importance of blood monitoring, and how users can mitigate risks by starting conservatively. Through this iterative process, the digital reviews ensured the final resource was comprehensive, grounded in community knowledge, and well-tailored to the needs of AAS-using communities.

### Implementation: where, when, who, how?

The health conversation tool is specifically designed for use by health workers who work in settings such as needle syringe provision when engaging with people who use AAS. This implementation section outlines the key considerations regarding the context, timing, personnel, and procedures associated with the application of this tool.

**Where**: The health conversation tool is intended for use in various health settings, particularly at needle and syringe programs and harm reduction services. These environments are ideal for reaching people who may benefit from tailored support related to AAS use.

**When**: The tool can be integrated into existing appointments or interactions, facilitating engagement during routine visits. Health workers can initiate the conversation during opportunistic encounters where AAS use is identified through screening and standard service provision. This flexible timing allows for personalised discussions tailored to the specific needs and contexts of the individual.

**Who**: The primary users of this tool are trained health workers, including outreach staff, counsellors, and peer workers at needle service providers. These individuals are equipped to guide discussions and provide evidence-based information.

**How**: The tool is presented as a hard copy resource that health workers can refer to during conversations. It serves as a guide to facilitate the discussion, ensuring that key topics and health information are effectively communicated. The tool can be given to people using AAS as a takeaway resource, providing them with valuable health information that they can refer to later.

At the conclusion of the conversation, health workers should determine the most appropriate course of action based on the persons circumstances. It is essential to establish guidelines for instances where it may not be appropriate to utilise the health conversation tool. For example, in acute care settings or crisis situations, the focus may need to shift away from structured conversations to immediate support and intervention. Health workers should exercise professional judgment to assess the appropriateness of implementing the tool in various contexts. We also note that this tool is not intended for robust data collection and recording, it serves as a guide.

## Discussion

This qualitative study focused on the co-production of a health conversation tool tailored for workers within health services (e.g., needle and syringe programs) to engage with people who use AAS. The collaborative design process was instrumental in shaping the final tool, ensuring that it directly reflected the lived-living experiences, concerns, and priorities of people who use AAS. By co-producing together as peers, we aimed to create a conversation that was not only relevant but also responsive to the specific needs and contexts of this underrepresented and marginalised community. Co-designing the health conversation tool with AAS peers was crucial for several reasons. First, it ensured that the language, tone, and content of the conversation resonated with people who use AAS, making it more likely that the community would engage with and trust the information being provided. Traditional harm reduction services often fail to reach people who use AAS because the materials and messaging are perceived as judgmental, irrelevant, or misaligned with their lived-living experiences [17]. By involving peers, we were able to co-create a conversation that attempts to foster an environment of mutual respect and shared understanding. The iterative feedback loop allowed for adjustments to ensure that the health conversation addressed these complexities and remained grounded in the lived-living experience and collective knowledge of the community. This is particularly important given recent research [39–41] which demonstrates that AAS consumers often encounter stigma, pathologisation, and contested notions of health in healthcare settings, making them hesitant to engage. By centring peer perspectives, the health conversation tool seeks to acknowledge these dynamics rather than reproducing the marginalisation observed in extant work.

This health conversation tool aligns with the core principles of harm reduction [82, 83] by empowering people who use AAS with information to make safer, informed choices without coercion or judgment [37]. Rather than adopting a punitive or prescriptive stance, the conversation is built around the premise of meeting people ‘where they are’ [84] —acknowledging the diverse reasons people may choose to use AAS and providing non-stigmatising guidance to reduce harm. Central to harm reduction is the concept of autonomy, where individuals are equipped with the knowledge and tools to make decisions about their own health [85]. The co-produced conversation embraces this philosophy, focusing on providing people with options for safer use practices, rather than mandating abstinence or imposing behavioural changes. However, it is important to recognise that informed decision-making does not always equate to safer or healthier outcomes. People may make well-informed choices that still involve risk, such as increasing AAS dosage beyond supraphysiological levels or combining several compounds (i.e., stacking). Within a harm reduction framework [85], this is not a failure of the approach but a recognition that autonomy and ‘being safer’ often coexist in tension [63]. The aim is not to ‘convince’ through moral argument but to enable a fuller understanding of potential harms, uncertainties, and alternatives. For instance, health workers can encourage delay among younger consumers as well as further reflection prior to initiation, or safer pathways without compromising respect for autonomy. This approach remains anchored in empathy and realism, acknowledging that informed choices can also include choosing not to begin using AAS at that moment in time. By fostering honest discussions about the potential risks, including long-term cardiovascular issues, mental health impacts, and gender-specific concerns like virilisation in women, this health conversation aims to offer a balanced perspective that respects a person’s choices.

### Implications

Despite the critical need for AAS checking services, their implementation is rare due to significant logistical and technical challenges [9]. These challenges include the need for costly off-site testing facilities, validated methods, reference standards, and specialised staff. Addressing these issues is essential to mitigate the health risks associated with illicit AAS use and provide necessary support to consumers. In Queensland, Australia the ROIDCheck program represents a significant development in harm reduction for people who use AAS. Initiated as a testing trial in 2024, ROIDCheck initially provided group-level results to participants [43, 44, 86]. By 2025, the program expanded to offer individualised testing results delivered alongside a tailored health conversation, which this paper reports the development of. AAS checking initiatives like are continuing to evolve both in Australia and internationally, for example, in Switzerland, where similar approaches have now been implemented [7]. Collectively, these programs mark important progress toward advancing health equity for people who use AAS worldwide.

Integrating this co-designed conversation into existing harm reduction services, beyond those offering AAS checking exclusively, represents an important step in expanding the scope of support available to AAS communities. Currently, harm reduction frameworks globally [1, 13, 15, 87, 88] have little to no infrastructure dedicated to addressing AAS consumption specifically. This health conversation tool bridges the current gap in harm reduction services for this population. Expanding the peer workforce is essential to ensuring the sustainability and effectiveness of harm reduction interventions tailored to people who use AAS. Currently, there is a significant gap in harm reduction services, not only in terms of the availability of AAS-specific resources but also in the number of trained peers who can deliver these interventions [55]. By increasing the peer workforce, harm reduction programs can become more responsive and accessible, especially to communities that are often marginalised or overlooked in mainstream healthcare settings.

Peers bring a unique and invaluable perspective to harm reduction, as they are able to establish trust and rapport more easily with people who use drugs due to their shared experiences [89–91]. This is particularly important in addressing the stigma and misconceptions surrounding AAS use, which can often prevent people from seeking traditional health services [27, 30, 49, 92, 93]. Expanding the peer workforce would not only enhance the reach of harm reduction efforts but also ensure that interventions remain grounded in the realities of those most affected. Moreover, peers are often uniquely positioned to offer context-specific and practically grounded advice that complements, rather than replaces, professional knowledge [39]. They can provide insights into the diverse motivations behind AAS use, including aesthetic, performance, and psychological factors, as well as tailor harm reduction messages to the specific needs of different subgroups within the AAS community, such as women and younger initiates. By training more peers, harm reduction services can be adapted to reflect the varying experiences and concerns of the broader AAS-using population, ensuring that interventions are not one-size-fits-all. Lastly, integrating more peers into the workforce also has the potential to create a feedback loop, where peers are continually engaged in refining and improving harm reduction strategies based on their ongoing interactions with the community. This iterative process would ensure that harm reduction conversations, like the one designed in this study, evolve alongside the changing trends, substances, and practices within AAS-using communities. Consequently, expanding the peer workforce is not just an operational necessity but a strategic imperative for long-term, effective harm reduction.

The success of this model also suggests broader applications beyond AAS. The collaborative, peer-driven design process could be replicated across different populations and substances, allowing harm reduction services to be more adaptive, community-focused, and responsive to the unique risks and concerns of various communities. Furthermore, by integrating peer-designed interventions into the broader harm reduction ecosystem, health services can increase their relevance and accessibility, ultimately contributing to improved public health outcomes. The potential for digital integration is another critical aspect of this model’s broader implications. Given the significant online presence and informal feedback mechanisms already in place for AAS consumers, future iterations of the tool could involve digital platforms, reaching a larger audience and offering real-time, peer-reviewed information.

### Limitations

One limitation of this study is that it relied on a relatively small group of peers, which may not fully capture the diversity of the broader AAS-using community. However, this was a deliberate choice, informed by earlier groundwork, including 25 in-depth interviews and extensive prior research, which laid a strong foundation of understanding. A smaller group of experienced peers allowed for more focused, open dialogue and facilitated a deeper, more nuanced discussion around the creation of the health conversation tool. Larger groups might have impeded the intimacy and trust necessary for meaningful collaboration, making the smaller group essential for achieving the study’s objectives. While the collaborative process ensured relevance to those involved, the specific needs and experiences of other subgroups, such as non-binary individuals, older consumers, or those from different cultural backgrounds, may not have been fully represented. Additionally, the study focused on developing a health conversation primarily for use in face-to-face settings, limiting its immediate applicability in digital or remote harm reduction services, which are becoming increasingly important. However, for the purpose of this conversation, to aid richer details from respondents, the face-to-face setting was critical. As with many peer-driven interventions, the scalability of this conversation may face challenges due to varying levels of peer training, engagement, and resources in different regions. Lastly, it is also important to acknowledge that grounding the tool in community knowledge, while central to its relevance and acceptability, may carry some points of contention. Community practices are not always aligned with best practices, and there is a risk that peer knowledge may reinforce partially accurate beliefs. Continued peer-led research is essential to ensure that future iterations of the tool maintain both authenticity and accuracy. These limitations suggest the need for future research to further refine and adapt the health conversation tool to be inclusive of a wider demographic and more flexible in its delivery methods.

## Conclusion

This study describes the development of a co-designed health conversation tool for health workers engaging with people who use AAS. The co-designed tool offers a novel, inclusive approach to addressing AAS-related harm, prioritising the voices and lived-living experiences of the community to ensure that interventions are not only relevant but effective. By aligning with harm reduction principles, the conversation empowers people to make informed, safer choices, while challenging the stigma that often accompanies AAS consumption. Its integration into existing harm reduction services could fill a significant gap, offering a model for future peer-driven interventions across various substance use contexts. The collaborative process itself underscores the importance of centring peer voices in drug policy and health intervention design, ensuring that services evolve in line with the needs and realities of the communities they serve.

## Supplementary Information

Below is the link to the electronic supplementary material.

Supplementary Material 1.

## Data Availability

Available from the corresponding author on reasonable request.
