# Supplementary material for: Co-producing a peer-led health conversation tool for the health services workforce to facilitate safer use of anabolic–androgenic steroids
Source: Harm Reduct J. 2025 Nov 24;22:202. doi: 10.1186/s12954-025-01356-z (PMC12750734; doi:10.1186/s12954-025-01356-z)
Supplement: Supplementary file 1 — Supplementary Material 1. [file 12954_2025_1356_MOESM1_ESM.docx]

**Health Worker Anabolic-Androgenic Steroid Conversation**

G’day mate, I want to discuss some important information to help you make more informed choices about your use.

1. Brief assessment
   1. Have you used image and performance enhancing drugs like anabolic-androgenic steroids before?
      1. Never
      2. A few times
      3. More than a few times
   2. How confident are you regarding your knowledge of these drugs?
      1. Not confident
      2. Confident
      3. Very confident
   3. Do you feel like you have adequate support around you to assist you with your health decisions?
      1. No – give me support
      2. Yes – but I want more
      3. Yes – I’m good, leave me be
2. What harm reduction strategies do you use when you're using?

| **Strategy** | **Know about this harm reduction strategy and use this harm reduction strategy (YES/NO)** | **Know about this HR strategy but don't use it [discuss + record reasons for not using this strategy]** | **Don't know about this HR strategy - tell me more:  YES/NO [with consent - provide the client with resources + info focused on the specific HR strategy that has been indicated]** | **Harm Reduction Advice (Brief)** |
| --- | --- | --- | --- | --- |
| Do you test your steroids or other image and performance enhancing drugs? |  |  |  | **IF NO:** Regular steroid checking is vital. Make sure you're using services to verify what you're taking (e.g., PEDTest Australia, CheQpoint in Queensland).  - https://www.quihn.org/cheqpoint/  - https://pedtestaustralia.com/ |
| Knowledge - Do you have access to information and resources to keep you safe(r) with your use? |  |  |  | **IF NO:** Please see digital literacy resources. **Safer Use Information:**   1. **Steroid-Specific Info:** I recommend checking out the steroid-specific information on Hi-Ground. They have excellent resources developed with the community. These resources cover everything from first-time use and safe injecting practices to the properties and half-lives of different compounds, common side effects, and where to get further support. Here are the links:    - https://hi-ground.org/substances/steroids/    - https://hi-ground.org/resource-types/steroid-resources/ |
| Have you ever received/performed regular health checks or regular health monitoring done (inc. bloodwork, and blood pressure)? |  |  |  | **IF NO:**  1**.** Getting regular bloodwork is crucial, either under in-person medical professional's supervision or through online services with medical consultation. This helps you monitor your health and catch any issues early.  - www.i-medical.com.au  - https://roidsafe.com.au/  - www.i-screen.com.au  2. Checking blood pressure weekly (or 2-3 times) and ensuring it does not stay elevated for prolonged periods (e.g., hypertensive category I, II,).   - **Blood Pressure Categories**:   - **Normal**: Systolic (the top number) is less than 120 mm Hg, and diastolic (the bottom number) is less than 80 mm Hg.   - **Elevated**: Systolic between 120 and 129 mm Hg, with diastolic less than 80 mm Hg.   - **Hypertension Stage 1**: Systolic between 130 and 139 mm Hg, or diastolic between 80 and 89 mm Hg.   - **Hypertension Stage 2**: Systolic 140 mm Hg or higher, or diastolic 90 mm Hg or higher. |
| Do you feel confident about how you administer your steroids?  (If injectable: are you performing safe injecting) (If oral – then do you know about liver stress) |  |  |  | **If Oral only:** If you're on an oral-only steroid cycle, keep it short to minimise liver stress.  **If injectable:** Do you have any injection sites red/swollen/infections/ etc.?  We have NSP here for your equipment needs and safe injecting resources available.  If both – provide both.  Link re: safe injecting:   - - https://hi-ground.org/substances/steroids/ |
| Do you have a usage strategy in place? |  |  |  | **IF NO:** If you are ‘cycling’, ensure that time ‘off’ really does equal time on, and make sure you have a post-cycle therapy (PCT) plan or taper.  **IF YES WOMEN:** Taper use down if possible.  See Hi-Ground resources.   - https://hi-ground.org/substances/steroids/ - https://hi-ground.org/resource-types/steroid-resources/ |
| Are you experiencing any physical or mental distress?  For example: hair loss or excessive growth, the development of breast tissue (gynecomastia) in men, changes in sexual function, acne, mood swings and bouts of depression, heightened aggression, paranoia, anxiety, sleep disturbances, menstrual cycle changes, virilisation (in women). |  |  |  | **IF YES:** Checking in with peers regarding mental health, re-assessing goals, reconsidering usage practices – see Hi-Ground for details.  Here are the links:   - https://hi-ground.org/substances/steroids/ - https://hi-ground.org/resource-types/steroid-resources/ |
